# Growth and physiological response of Yulu *Hippophae rhamnoides* to drought stress and its omics analysis

**DOI:** 10.1080/15592324.2024.2439256

**Published:** 2024-12-09

**Authors:** Haipeng Chen, Xiaolin Chen, Xiaogang Li, Xin Lin, Lihua Yue, Chunhai Liu, Yuling Li

**Affiliations:** a College of Forestry, Hebei Agricultural University, Baoding, Hebei, China; b Technical Center, Chengde Astronaut Mountainous Plant Technology Co. Ltd. Chengde, Hebei, China

**Keywords:** Drought stress, Yulu *Hippophae rhamnoides*, root system, differential gene, differential protein

## Abstract

*Hippophae rhamnoides (H. rhamnoides*) is the primary tree species known for its ecological and economic benefits in arid and semi-arid regions. Understanding the response of *H. rhamnoides* roots to drought stress is essential for promoting the development of varieties. One-year-old Yulu *H. rhamnoides* was utilized as the experimental material, and three water gradients were established: control (CK), moderate (T1) and severe (T2), over a period of 120 days. The phenotypic traits and physiological indies were assessed and analyzed, while the roots were subjected by RNA-Seq transcriptome and Tandem Mass Tags (TMT) proteome analysis. Drought stress significantly reduced the plant height, ground diameter, root biomass and superoxide dismutase activity; however, the main root length increased. In comparison with CK, a total of 5789 and 5594 differential genes, as well as 63 and 1012 differential proteins, were identified in T1 and T2, respectively. The combined analysis of transcriptome and proteome showed that the number of differentially expressed genes (DEGs) and differentially expressed proteins (DEPs) associated with T1, T2 and CK was 28 and 126, respectively, with 7 and 36 genes achieving effective KEGG annotation. In T1 and T2, the differential genes were significantly enriched in the plant hormone signal transduction pathway, but there was no significant enrichment in the protein expression profile. In T2, 38 plant hormone signal transduction function genes and 10 peroxisome related genes were identified. With the increase of drought stress, the combined expression of DEGs and DEPs increased. Yulu *H. rhamnoides* may allocate more resources toward CAT while simultaneously decreasing SOD and POD to mitigate the oxidative stress induced by drought. Furthermore, the molecular mechanisms underlying plant hormone signal transduction and peroxisome-related genes in the roots of *H. rhamnoides* were discussed in greater detail.

## Introduction

Drought stress usually has an adverse effect on plant growth,^1^ resulting in delayed growth,^2^ abnormal flowering,^3^ blocked photosynthesis^4^ and oxidative stress,^5^ and can cause irreversible damage to plant tissues.^6^ Plants will undergo a series of adaptive changes under drought stress. For example, plants will reduce transpiration tension by adjusting stomatal opening and closing to reduce water loss.^7–9^ Concurrently, plants can increase the concentration of leaf cell sap, enhancing the content of proline and other solutes to lower osmotic potential and improve water retention capacity, thereby employing various physiological mechanisms to cope with drought stress.^10,11^ At the molecular level, plants express a series of genes related to drought stress,^12^ encoding drought-related proteins such as transcription factors, protein kinases, and hydrolases, to regulate and respond to the molecular mechanism associated with drought stress.^13^ Many xerophytes have developed diverse protective mechanisms to withstand extreme conditions and produce genes that confer resistance to environmental stress.^14^ Therefore, understanding the molecular basis of xerophytes’ adaption to drought can provide a theoretical foundation for further research on the drought tolerance mechanism in plants.

*Hippophae rhamnoides* exhibits characteristics such as drought tolerance, cold tolerance and resistance to wind and sand. Its well-developed root system possesses nitrogen-fixing capabilities, which can adapt to arid and barren soil.^15–17^ *H. rhamnoides* is widely used in the greening of degraded land such as sandy land. It is a pioneer tree species for controlling soil erosion, curbing desertification and improving ecological environment in arid and semi-arid areas.^18,19^ At the same time, *H. rhamnoides* has the same value of medicine and food, and its economic value is considerable.^20^ In addition to fresh food, *H. rhamnoides* fruit can also be processed into fruit juice, fruit wine, and jam.^21^ *H. rhamnoides* oil can also be used to make health care products and cosmetics.^22^ With the further development of *H. rhamnoides* industry, it will drive the development of *H. rhamnoides* planting industry, processing industry and sales industry chain in mountainous areas, and increase the income of farmers. *Hippophae rhamnoides subsp. sinensis ‘Yulu’* is a new variety bred by Chengde Astronaut Mountainous Plant Technology Co. Ltd. It is characterized by high oil, high yield and medium-sized fruit, which has great ecological and economic value. It has been included in the recommended catalog of afforestation, grass, forest and grass varieties in China’s ‘Three North’ project.

When subjected to drought stress, *H. rhamnoides*, like many drought-resistant plants, employs various strategies to mitigate its effects, including morphological changes, differential gene expression, and the regulation of physiological mechanisms and hormone signal stress response.^23^ *H. rhamnoides* has strong drought resistance and water retention capabilities, with its root system playing an important role in the drought resistance process of *H. rhamnoides*.^24^ Plant roots can increase the specific surface area and root surface area under drought stress, which helps to improve their water absorption capacity.^25^ Under drought stress, osmotic adjustment substances such as proline and soluble sugar in plant roots accumulate, thereby increasing the osmotic adjustment ability of cells and tissues. These substances play an important role in maintaining cell turgor and preventing water loss.^26^ Furthermore, the antioxidant system of plant roots can also effectively remove reactive oxygen species produced under drought stress and reduce oxidative damage.^27^ With the development of sequencing technology and the assembly of plant genomes, researchers have begun to reveal the mechanism of *H. rhamnoides* in response to drought stress from the level of gene expression. However, most of them have analyzed the gene level of the aboveground part of *H. rhamnoides*,^23^ and the use of multi-omics technology for *H. rhamnoides* roots under drought stress is unclear.

In this study, the growth, physiological, and biochemical indexes of *H. rhamnoides* under different drought stress conditions were measured, and the transcriptome and proteome of *H. rhamnoides* roots were sequenced to analyze the phenotypic, physiological, and biochemical response characteristics of *H. rhamnoides* under drought stress. On this basis, the possible biological functions of differentially expressed genes (DEGs) and differentially expressed proteins (DEPs) in *H. rhamnoides* roots under drought stress were analyzed, and their potential effects on *H. rhamnoides* drought stress were evaluated. The results provide a reference for further study of the regulatory network of *H. rhamnoides* under drought stress.

## Materials and methods

### Testing material

In this study, the one-year-old cutting seedlings of Yulu *H. rhamnoides* (Y) from Chengde Astronaut Mountainous Plant Technology Co. Ltd. (Chengde, Hebei, China) were selected as the research objects. At the end of April 2021, Yulu *H. rhamnoides* (with an average plant height of 16.08 cm and an average ground diameter of 3.85 mm) exhibiting similar growth vigor were selected as the test plant. These seedlings were planted in nutrient bowls measuring 50 cm × 50 cm, using river sand as the substrate. The total nitrogen, total phosphorus and total potassium were 0.82 g/kg, 0.89 g/kg, and 5.53 g/kg, respectively, and the alkali-hydrolyzed nitrogen, available phosphorus and available potassium were 14.12 mg/kg, 6.47 mg/kg, and 32.73 mg/kg, respectively.

The experiment was carried out in the teaching experimental farm of Hebei Agricultural University (Baoding, Hebei, China). The potted water control experiment was utilized to simulate drought stress experiment. When the actual soil moisture content was about 10%, *H. rhamnoides* was still able to maintain a healthy growth state. Therefore, the experimental design aimed to impose a more arid soil environment, with actual soil moisture content at 11.5% ~ 7.5% (CK), 7.5% ~ 4.5% (T1), 4.5% ~ 1.5% (T2).and 75 pots were planted under each water condition, resulting in 225 pots overall. The drought stress treatment began in late June 2021 and ended in late October, spanning a total duration of 120 days. T1 and T2 indicated moderate and severe stress, respectively. At the end of the stress, the whole harvest method was used to collect roots for the extraction of total RNA and protein from roots, as well as the determination of root growth, physiological, and biochemical parameters. Following sample collection, the specimens were immediately frozen in liquid nitrogen and stored at −80°C.

### Growth, physiological and biochemical determination

In order to understand the changes in growth, root physiological and biochemical parameters of *H. rhamnoides* under drought stress, measurements were taken at the conclusion of the drought stress test. Specifically, the plant height and ground diameter were measured for 30 *H. rhamnoides* individuals. Additionally, the root length, root water content and aboveground and root biomass were measured for 3 *H. rhamnoides* individuals. And the total biomass and root:shoot ratio were calculated. The root:shoot ratio was calculated as the ratio of root biomass to above ground biomass. The root peroxidase (POD) activity, superoxide dismutase (SOD) activity and catalase (CAT) activity were measured and repeated six times. Biomass and relative water content were determined by drying method,^28^ POD was determined by guaiacol method,^29^ CAT was determined by ultraviolet absorption method,^30^ and SOD was determined by nitrogen blue tetrazolium method.^31^

### Transcriptome analysis

The RNA extraction, library construction, quality inspection and RNA sequencing of root tissue from *H. rhamnoides* were conducted using equipment from Novogene Company (Beijing, China, https://cn.novogene.com/).

The cDNA library construction and de novo transcriptome assembly were completed by Novogene equipment (Beijing, China). Three RNA samples with different drought levels (with three biological replicates per sample) were used for cDNA library construction. Following the completion of the library construction, the Qubit 2.0 Fluorometer was used for preliminary quantification, and then Agilent 2100 bioanalyzer was used to detect the insert size of the library. After the insert size was in line with expectations, qRT-PCR was conducted to accurately quantified the effective concentration of the library (the effective concentration of the library was higher than 2 nM) to ensure the quality of the library. After qualifying the library, the different libraries were pooled based on effective concentration and the target offline data volume. Sequencing was then performed using the Illumina NovaSeq 6000, resulting in the generation of 150 bp paired-end reads. Then the raw data was filtered to obtain clean reads for transcriptomics analysis.

Due to the absence of a classical reference genome, Trinity (v2.6.6) software was used to de novo assemble the clean reads, thereby generating a reference sequence for subsequent analysis. The longest Cluster sequence was obtained by Corset hierarchical clustering for subsequent analysis. BUSCO software was used to evaluate the quality of the assembly.^32,33^ In order to obtain comprehensive gene function information, seven databases were annotated for gene function, including: Nr, Nt, Pfam, KOG/COG, Swiss-prot, KEGG and GO.^34–36^ Differential expression analysis of the two groups was performed using DESeq2 (1.20.0). The criteria for screening differentially expressed genes was |log2(FoldChange)| > 1 and padj < 0.05.^37^ The statistical power of this experimental design, calculated in Power analysis (https://rodrigo-arcoverde.shinyapps.io/rnaseq_power_calc/) is 0.9999693, 0.9989935 and 0.9998026.

### Proteomic analysis

The preparation of *H. rhamnoides* root tissue and the subsequent quantitative proteomics analysis were completed by Novogene Company (Beijing, China, https://cn.novogene.com/) using Tandem Mass Tags (TMT) technology to label quantitative proteome. *H. rhamnoides* root samples were lysed and protein extracted by SDT protein lysate, dithiothreitol (DTT, purchased from Sigma/D9163-25 G), iodoacetamide (IAM, purchased from Sigma/I6125-25 G) and acetone.^38–42^ Protein quantification was performed with the Bradford protein quantification kit. Following this, protein digestion and desalination, TMT labeling, fraction separation, and mass spectrometry detection were executed.^43–45^ Details were shown in Supporting Information.

Based on the raw data obtained from mass spectrometry detection, the software Proteome Discoverer_2.4 was used to search the corresponding database (mainly based on transcriptome prediction proteins). Protein identification was subsequently conducted based on the results of database search. At the same time, the mass tolerance distribution of peptides, proteins, and parent ions was analyzed to evaluate the quality of mass spectrometry detection data. The identified proteins were annotated using common functional databases, including COG, GO and KEGG databases.^46–50^ The criteria for screening differential proteins were as follows: when FC ≥ 1.2 and *p* value ≤ 0.05, up-regulated proteins were screened; when FC ≤ 0.83 and *p* value ≤ 0.05, down-regulated proteins were screened.

### Association analysis of transcriptome and proteome

The basic principle of joint analysis of proteome and transcriptome is the central principle. The correlation between protein IDs and gene names, as well as between transcription IDs and gene names, indicates an indirect relationship between protein IDs and transcription IDs. Building on this premise, we conducted a comparative analysis of the differences between the two. We performed an overlap analysis of the gene name sets obtained from identified proteins (transcriptions) and differential proteins (transcriptions), revealing the correlation between the identified expression levels and the differential expression levels of both. Subsequently, we compared and displayed the two omics-related proteins and their transcriptional fold changes (FC), examining both their differences and commonalities. By combining the GO and KEGG results, we explored the functional or metabolic pathway similarities and differences. Finally, based on the results of protein enrichment, the expression of the associated transcriptional IDs was compared with the differentially expressed proteins.^51^

### Data analysis

Physiological and biochemical data were analyzed using SPSS 24 software (SPSS, USA) and Microsoft Office 2016 (Microsoft, USA) for variance analysis. Duncan’s multiple range test was used to detect the difference between the mean values at the significance level of *p* < 0.05.

## Results

### *Phenotypic and physiological responses of Yulu* H. rhamnoides *to drought stress*

With the increase of drought degree, the plant height, ground diameter, total biomass, root biomass, aboveground biomass, root: shoot ratio and root relative water content of *H. rhamnoides* showed a decreasing trend, however, the main root length showed an increasing trend (Table 1). Compared with CK, plant height, ground diameter, total biomass, root biomass and root relative water content in T2 decreased significantly (*p* < 0.05), while only the plant height in T1 decreased significantly (*p* < 0.05).

**Table 1.** Phenotypic status and physiological indicators of yulu *hippophae rhamnoides* under drought treatment.

Treatment	CK	T1	T2
Plant height/cm	97.30 ± 18.39a	82.52 ± 13.18b	62.97 ± 13.75c
Ground diameter/mm	8.85 ± 1.42a	8.32 ± 0.99a	6.65 ± 0.95b
Biomass/g	66.48 ± 31.51a	53.72 ± 7.69ab	20.70 ± 2.31b
Root biomass/g	40.71 ± 20.24a	31.89 ± 10.84ab	9.74 ± 1.01b
Above ground biomass/g	79.82 ± 42.34a	72.74 ± 9.15a	33.22 ± 8.16a
Main root length/cm	104.33 ± 24.58a	114.67 ± 31.57a	129.67 ± 11.68a
root:shoot ratio	1.56 ± 0.19a	1.54 ± 0.79a	0.90 ± 0.17a
Root water content/g	183.07 ± 74.90a	170.28 ± 24.36a	56.88 ± 5.22b
CAT U/mg FW	55.70 ± 49.11a	63.52 ± 33.47a	89.5 ± 64.51a
POD U/mg FW	17.88 ± 25.68a	15.07 ± 9.32a	9.15 ± 7.59a
SOD U/g FW	200.19 ± 63.20a	132.20 ± 45.34b	109.89 ± 38.99b

CK: control, T1: moderate stress, T2: severe stress. Different lowercase letters indicated that there was a significant difference between different treatments (*p* < 0.05).

It can be seen that under different water gradients, the phenotypic traits of Yulu *H. rhamnoides* were significantly different, and the root biomass was significantly reduced, indicating that the root traits changed more significantly. Furthermore, the key enzymes of the three enzymatic defense systems – catalase activity (CAT), peroxidase activity (POD) and superoxide dismutase activity (SOD) – in the roots of *H. rhamnoides* were determined and analyzed. The results are shown in Table 1. With the increase of drought degree, SOD and POD in root decreased gradually, and CAT in root increased gradually, but the difference was not significant (*p* > 0.05). Notably,SOD in T1 and T2 was significantly lower than that in CK (*p* < 0.05).

The results of the phenotypic and physiological indexes showed that *H. rhamnoides* exhibited pronounced stress response characteristics after 120 days of drought. Plant height, ground diameter, root biomass, root relative water content, and SOD activity decreased significantly. In order to further investigate the molecular mechanisms underlying the drought stress response in *H. rhamnoides*, we selected the roots of *H. rhamnoides* for transcriptome and proteome sequencing, and analyzed the regulatory network of *H. rhamnoides* roots in response to drought stress.

### *Transcriptome quality analysis and DEGs identification of* H. rhamnoides *roots*

Three sets of biological replicate sequences were established for each treatment, and nine cDNA libraries (Y_CK_1,_2,_3 and Y_T1_1,_2,_3 and Y_T2_1,_2,_3) were constructed. After sequencing on the illumina NovaSeq 6000 platform(illumina, USA), 63.4 Gb clean data was obtained. The clean read ratio of 9 samples was 95.38 ~ 96.65% (Table S1). The clean data of each sample exceeded 6.0 Gb, with Q20 ≥ 97.4%, Q30 ≥ 93.2%, and GC content in all samples greater than 42%. The results showed that the quality of the transcriptome data was high enough for subsequent analysis.

After Trinity assembly, the total number of nucleotides spliced with Transcript was 279,123,596, while the total number of nucleotides spliced with Unigene was 74,563,702. The N50 for Transcript and Unigene were 2528 and 2319, respectively, and the assembly integrity was high (Table S2).

**Figure 1.**
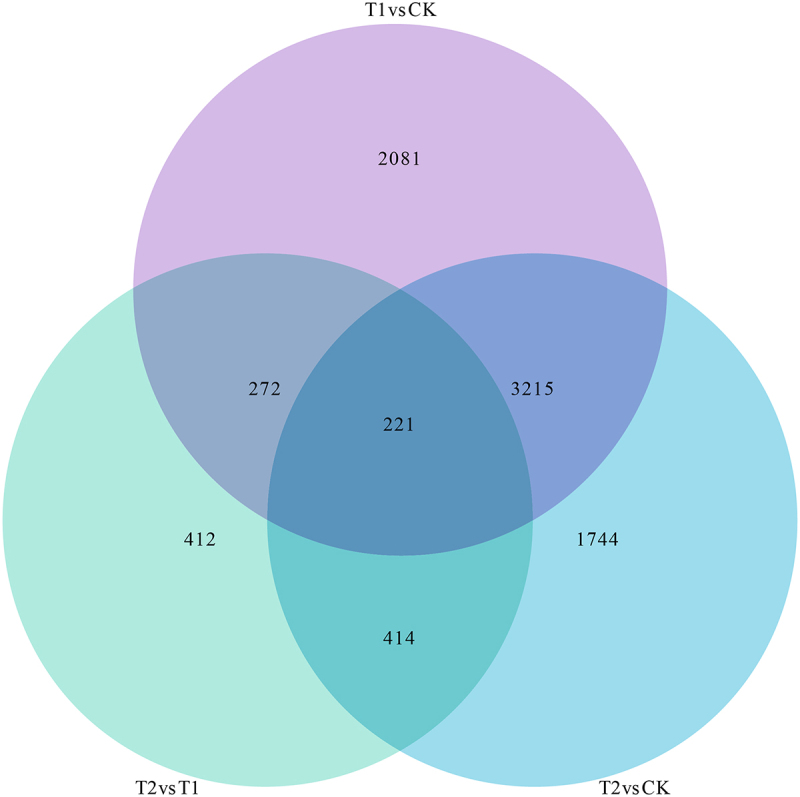
Transcriptome differential gene statistics of Yulu *Hippophae rhamnoides.*

Using the criteria of DESeq2 with pval < 0.05 and |log2FoldChange|>0, DEGs between treatments were screened, and the results are shown in Table S3. Compared with CK, T1 obtained 5789 DEGs, of which 2739 were up-regulated and 3050 were down-regulated. A total of 5594 DEGs were obtained in T2 compared with CK, of which 2536 were up-regulated and 3058 were down-regulated. A total of 1319 DEGs were obtained between T2 and T1, of which 472 were up-regulated and 847 were down-regulated. Among the DEGs, 221 were common to all comparisons (T1 vs CK, T2 vs CK, and T2 vs T1), while 2081 DEGs were exclusive to T1 vs CK, 1744 DEGs were unique to T2 vs CK, and 412 DEGs were specific to T2 vs T1 (Figure 1).

### *Proteome sequencing and DEPs identification of* H. rhamnoides *roots*

The results of the quantitative proteomics analysis, based on TMT labeling of the roots of Yulu *H. rhamnoides*, are presented in Table S4. In nine samples, the total number of secondary spectra was 232,180, the effective number of spectra was 24,180. A total of 16,961 peptides were identified, the number of identified proteins was 5345, and the number of quantifiable proteins was 5326.

When FC ≥ 1.2 and *p* value ≤ 0.05, the up-regulated proteins were screened. When FC ≤ 0.83 and *p* value ≤ 0.05, the down-regulated proteins were screened. The results are shown in Table S5. A total of 63 DEPs were obtained from T1 compared with CK, of which 32 were up-regulated and 31 were down-regulated. Compared with CK, 1012 DEPs were obtained in T2, of which 381 were up-regulated and 631 were down-regulated. A total of 581 DEPs were obtained between T2 and T1, of which 233 were up-regulated and 348 were down-regulated.

### Functional enrichment analysis of DEGs

**Figure 2.**
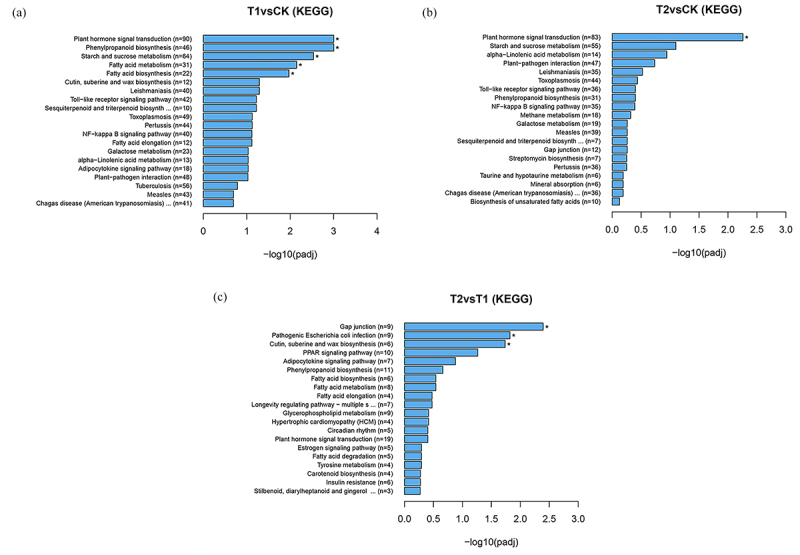
KEGG pathways enrichment analysis of DEGs of Yulu *Hippophae rhamnoides*. a) KEGG pathways enrichment analysis of T1vsCK DEGs of Yulu *Hippophae rhamnoides*. b) KEGG pathways enrichment analysis of T2vsCK DEGs of Yulu *Hippophae rhamnoides*. c) KEGG pathways enrichment analysis of T2vsT1 DEGs of Yulu *Hippophae rhamnoides*.

The enrichment of identified DEGs in the KEGG pathways under varying degrees of drought stress is illustrated in Figure 2.The main metabolic pathways associated with the differential genes between T1 and CK include plant hormone signal transduction (90 DEGs), phenylpropanoid biosynthesis (46 DEGs), starch and sucrose metabolism (64 DEGs), fatty acid metabolism (31 DEGs), fatty acid biosynthesis (22 DEGs), cutin, suberine and wax biosynthesis (12 DEGs), sesquiterpenoid and triterpenoid biosynthesis (10 DEGs), toll-like receptor signaling pathway (42 DEGs). Notably, the pathways of plant hormone signal transduction, phenylpropanoid biosynthesis, starch and sucrose metabolism, fatty acid metabolism, fatty acid biosynthesis were significantly enriched (Figure 2a). These findings indicate that, compared with CK, these DEGs in T1 were mainly involved in root signal transduction, secondary metabolite biosynthesis, carbohydrate metabolism, and lipid metabolism.

The main metabolic pathways associated withDEGs between T2 and CK include plant hormone signal transduction (83 DEGs), starch and sucrose metabolism (55 DEGs), alpha-linolenic acid metabolism (14 DEGs), plant-pathogen interaction (47 DEGs), phenylpropanoid biosynthesis (3 DEGs), toll-like receptor signaling pathway (36 DEGs), NF-kappa B signaling pathway (35 DEGs), and methane metabolism (18 DEGs). Notably, plant hormone signal transduction is a significantly enriched pathway (Figure 2b). These findings indicate that in T2, compared with CK, are primarily involved in root signal transduction, carbohydrate metabolism, lipid metabolism, environmental adaptation, and biosynthesis of secondary metabolites.

The main metabolic pathways associated with DEGs in T2 and T1 include gap junction (9 DEGs), cutin, suberine and wax biosynthesis (6 DEGs), PPAR signaling pathway (10 DEGs), adipocytokine signaling pathway (7 DEGs), phenylpropanoid biosynthesis (11 DEGs), fatty acid metabolism (8 DEGs), fatty acid biosynthesis (6 DEGs), longevity regulating pathway-multiple species (7 DEGs). Notably, the biosynthesis of gap junction, cutin, suberine and wax biosynthesis represents a significantly enriched pathway (Figure 2c). By comparing T2 to T1, these DEGs mainly responded to the root cell community, lipid metabolism, endocrine system, biosynthesis of secondary metabolites, and longevity regulation pathway in *H. rhamnoides*.

By comparing the KEGG enrichment of the three groups of differential genes, it was found that the common pathways among them included plant hormone signal transduction and phenylpropane biosynthesis. This suggests that under drought stress, the hormone regulation and enzymatic reaction in *H. rhamnoides* were extremely active, leading to adjustments in its growth strategy, such as stomatal opening and closing and osmotic pressure regulation to cope with drought stress.

**Figure 3.**
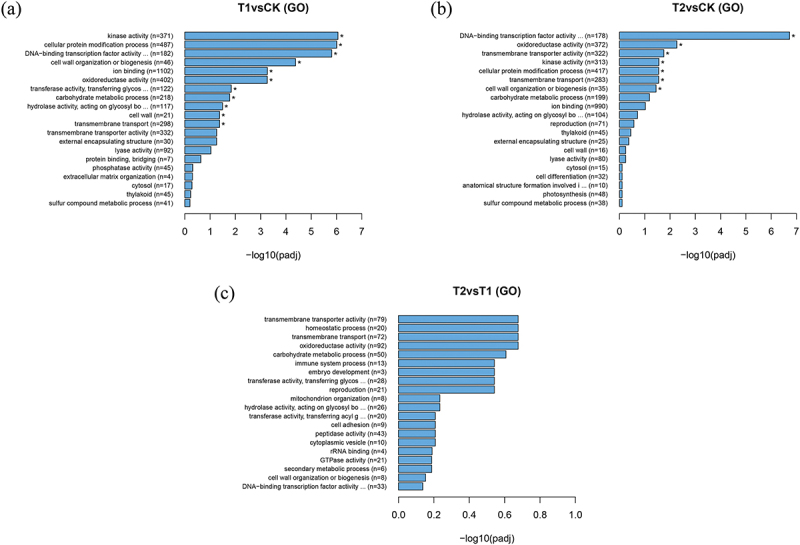
GO categorization of DEGs of Yulu *Hippophae rhamnoides*. a) GO categorization of T1vsCK DEGs of Yulu *Hippophae rhamnoides*. b) GO categorization of T2vsCK DEGs of Yulu *Hippophae rhamnoides*. c) GO categorization of T2vsT1 DEGs of Yulu *Hippophae rhamnoides*.

The function of DEGs was further elaborated by the enrichment of differential genes in GO (Figure 3). When comparingT1 with CK, the DEGs exhibited significant enrichment in several categories, including kinase activity (GO: 0016301,371 DEGs), cellular protein modification process (GO: 0006464,487 DEGs), DNA-binding transcription factor activity (GO:0003700, 182 DEGs), cell wall organization or biogenesis (GO: 0071554,46 DEGs), ion binding (GO: 0043167, 1102 DEGs), oxidoreductase activity (GO: 0016491,402 DEGs), transferase activity, transferring glycosyl groups (GO: 0016757,122 DEGs), carbohydrate metabolic process (GO:0005975, 218 DEGs), hydrolase activity,acting on glycosyl bonds (GO: 0016798,117 DEGs), cell wall (GO:0005618, 21 DEGs), and transmembrane transport (GO: 0055085, 298 DEGs) (Figure 3a). These functions encompass the roles of various proteins and enzymes, including phosphorylation and glycosylation of specific substances, which influence electron transfer, transmembrane movement of substances, as well as impacting cell shape and structure.

The DEGs between T2 and CK were significantly enriched in several functional categories, including DNA-binding transcription factor activity (GO:0003700, 178 DEGs), oxidoreductase activity (GO:0016491, 372 DEGs), transmembrane transporter activity (GO:0022857, 322 DEGs), kinase activity (GO:0016301, 313 DEGs), cellular protein modification process (GO:0006464, 417 DEGs), transmembrane transport (GO:0055085, 283 DEGs), and cell wall organization or biogenesis (GO:0071554, 35 DEGs) (Figure 3b). These functions are crucial for regulating gene expression, facilitating redox reaction, mediating protein exchange across the cell membrane, enabling cell signal transduction, and managing metabolic regulation. Additionally, they influence the structure, function, and stability of the cell wall and proteins.

Compared with T1, the enrichment of DEGs in T2 did not reach a significant level in GO. However, the genes were primarily enriched in several functional categories, including transmembrane transporter activity (GO:0022857, 79 DEGs), homeostatic process (GO:0042592, 20 DEGs), transmembrane transport (GO:0055085, 72 DEGs), oxidoreductase activity (GO:0016491, 92 DEGs), carbohydrate metabolic process (GO:0005975, 50 DEGs), immune system process (GO:0002376, 13 DEGs), embryo development (GO:0009790, 3 DEGs), transferase activity, transferring glycosyl groups (GO:0016757, 28 DEGs), reproduction (GO:0000003, 21 DEGs) (Figure 3c).

The comparison of GO enrichment of the three groups of DEGs revealed significant enrichment in processes such as oxidoreductase activity, transmembrane transport, and carbohydrate metabolism. These functions played an important role in the response of *H. rhamnoides* to drought stress, subsequently influencing the regulation of enzyme activity, water regulation, material distribution, and energy balance, thereby enhancing the resistance of *H. rhamnoides* to drought stress.

### Functional enrichment analysis of DEPs

**Figure 4.**
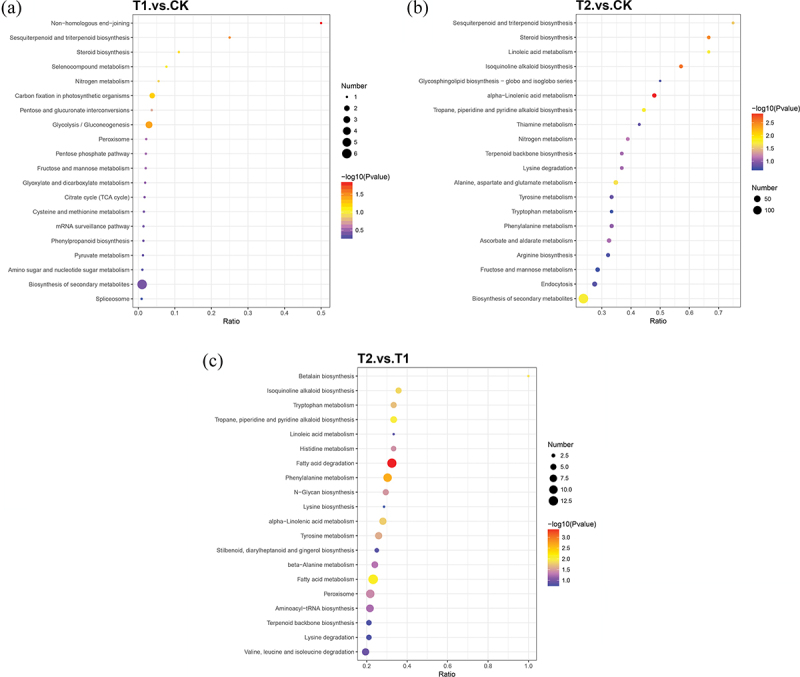
KEGG pathways enrichment analysis of DEPs of Yulu *Hippophae rhamnoides*. a) KEGG pathways enrichment analysis of T1vsCK DEPs of Yulu *Hippophae rhamnoides*. b) KEGG pathways enrichment analysis of T2vsCK DEPs of Yulu *Hippophae rhamnoides*. c) KEGG pathways enrichment analysis of T2vsT1 DEPs of Yulu *Hippophae rhamnoides*.

Under different degrees of drought stress, the enrichment of identified DEPs in KEGG is shown in Figure 4. The main metabolic pathways associated with DEPs between T1 and CK include non-homologous end-joining (NHEJ), sesquiterpenoid and triterpenoid biosynthesis, steroid biosynthesis, selenocompound metabolism, nitrogen metabolism, carbon fixation in photosynthetic organisms, pentose and glucuronate interconversions, glycolysis/gluconeogenesis, Peroxisome (Figure 4a). It can be seen that the DEPs in T1, compared with CK, are mainly involved in DNA breakage repair in the roots of *H. rhamnoides*, influencing the decomposition and synthesis of glucose, participating in the metabolic pathways of various compounds, and affecting the oxidation of substances as well as the metabolism of hydrogen peroxide.

The main metabolic pathways involved in the DEPs of T2 and CK include sesquiterpenoid and triterpenoid biosynthesis, steroid biosynthesis, linoleic acid metabolism, isoquinoline alkaloid biosynthesis, glycosphingolipid biosynthesis-globo and isoglobo series, alpha-linolenic acid metabolism, tropane, piperidine and pyridine alkaloid biosynthesis, thiamine metabolism and nitrogen metabolism (Figure 4b). Compared to CK, T2 exhibits differential proteins that predominantly reflect the circulation and metabolism of acids, alkaloids, alcohols, terpenoids, and root nitrogen in the roots of *H. rhamnoides*. These pathways and physiological are fully mobilized to enhance resistance to drought stress.

The main metabolic pathways associated with DEPs in T2 and T1 included betalain biosynthesis,isoquinoline alkaloid biosynthesis, tryptophan metabolism, tropane, piperidine and pyridine alkaloid biosynthesis, linoleic acid metabolism, histidine metabolism, fatty acid degradation, phenylalanine metabolism (Figure 4c). A comparison between T2 and T1 revealed that these DEPs mainly reflected in the metabolic pathways of fatty acids, alkaloids and various amino acids in the roots of *H. rhamnoides*. This suggests that the plant responds to water scarcity by modulating the synthesis and degradation of these compounds.

By comparing the KEGG enrichment of the three groups of DEPs, it was found that DEPs play an important role in various metabolic pathways, including fatty acids, alkaloids, amino acids, and nitrogen cycle. The modulation of these pathways aids *H. rhamnoides* roots in preserving the integrity and stability of cell structure, enhancing their defensive capabilities, and mitigating physiological changes caused by drought stress. This may also explain why physiological indicators exhibit minimal difference in response to drought stress.

### Combined analysis of proteome and transcriptome

**Figure 5.**
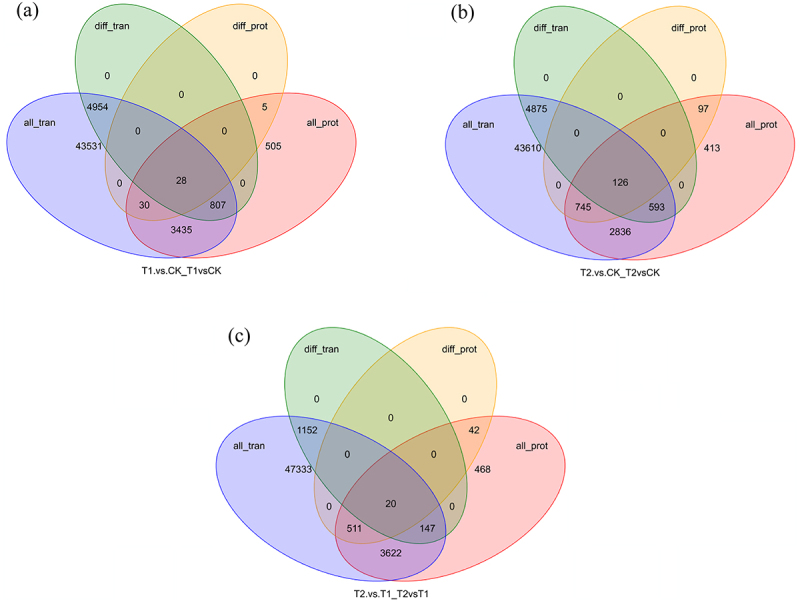
Transcriptome and proteome expression regulation. a) Gene and protein association diagram of T1vsCK. b) Gene and protein association diagram of T2vsCK. c) Gene and protein association diagram of T2vsT1. All_tran: all genes identified by the transcriptome; diff_tran: differentially expressed genes identified by the transcriptome; all_pro: all proteins identified by the proteome; diff_prot: differentially expressed proteins identified by the proteome.

In order to explore the relationship between DEPs and DEGs, we conducted a combined analysis of proteome and transcriptome using sequencing data of the roots of Yulu *H. rhamnoides*. The results of combined analysis are shown in Figure 5. We identified 28 associations between the T1 and CK differential genes and proteins, 126 associations between the T2 and CK differential genes and proteins, and 20 associations between the T2 and T1 differential genes and proteins. These genes were annotated by KEGG, yielding effective results of 7, 36 and 4, respectively (Table 2).

**Table 2.** Associated genes statistics table of KEGG pathways of Yulu *Hippophae rhamnoides.*

Treatment	KEGG pathways	Gene
T1vsCK	Steroid biosynthesis(map00100)	1
T1vsCK	Selenocompound metabolism(map00450)	1
T1vsCK	Protein processing in endoplasmic reticulum(map04141)	1
T1vsCK	Pentose and glucuronate interconversions(map00040)	1
T1vsCK	mRNA surveillance pathway(map03015)	1
T1vsCK	Glycolysis/Gluconeogenesis(map00010)	1
T1vsCK	Amino sugar and nucleotide sugar metabolism(map00520)	1
T2vsCK	Ubiquitin mediated proteolysis(map04120)	1
T2vsCK	Steroid biosynthesis(map00100)	2
T2vsCK	RNA transport(map03013)	1
T2vsCK	Plant-pathogen interaction(map04626)	2
T2vsCK	Peroxisome(map04146)	1
T2vsCK	Other glycan degradation(map00511)	1
T2vsCK	Metabolic pathways(map01100)	5
T2vsCK	Isoquinoline alkaloid biosynthesis(map00950)	2
T2vsCK	Endocytosis(map04144)	2
T2vsCK	Biosynthesis of secondary metabolites(map01110)	14
T2vsCK	Ascorbate and aldarate metabolism(map00053)	2
T2vsCK	alpha-Linolenic acid metabolism(map00592)	3
T2vsT1	Stilbenoid, diarylheptanoid and gingerol biosynthesis(map00945)	1
T2vsT1	Phenylpropanoid biosynthesis(map00940)	1
T2vsT1	Phenylalanine metabolism(map00360)	1
T2vsT1	Glutathione metabolism(map00480)	1

CK: control, T1: moderate stress, T2: severe stress.

Seven genes associated with DEPs and DEGs between T1 and CK were involved in the following pathways: steroid biosynthesis (map00100), selenocompound metabolism (map00450), protein processing in endoplasmic reticulum (map04141), pentose and glucuronate interconversions (map00040), mRNA surveillance pathway (map03015), glycolysis/gluconeogenesis (map00010), amino sugar and nucleotide sugar metabolism (map00520) (Table 2).

The 36 genes associated with DEPs and DEGs between T2 and CK are involved in 12 pathways. Among these, biosynthesis of secondary metabolites (map01110) brings 14 genes, metabolic pathways (map01100) consists of 5 genes, alpha-linolenic acid metabolism (map00592) comprises 3 genes, and steroid biosynthesis (map00100) contains 2 genes. In addition, there is an associated gene involved in the peroxisome (map04146) pathway (Table 2).

Four genes associated with DEPs and DEGs between T2 and T1 were involved in the following pathways: stilbenoid, diarylheptanoid and gingerol biosynthesis (map00945), phenylpropanoid biosynthesis (map00940), phenylalanine metabolism (map00360), glutathione metabolism (map00480) (Table 2).

### Screening of key genes for drought stress

The DEGs between T1 and CK, as well as T2 and CK, were significantly enriched in the plant hormone signal transduction pathway. Consequently, we conducted an analysis of the DEGs within this pathway. Given the substantial changes observed in the growth and omics indices of *H. rhamnoides* under severe drought stress, we screened for key genes associated with drought resistance based on the expression of DEGs between T2 and CK in plant hormone signal transduction pathway. Our focus was single up-regulated or single down-regulated differential genes, resulting in the identification of 38 genes related to plant hormone signal transduction (Table 3). This included 7 genes associated with auxin signal transduction, 4 with cytokinin signal transduction, 2 with gibberellin signal transduction, 6 with abscisic acid signal transduction, 5 with ethylene signal transduction, 2 with brassinosteroid signal transduction, 10 with jasmonic acid signal transduction, and 2 with salicylic acid signal transduction.

**Table 3.** *Hippophae rhamnoides* plant hormone signal transduction channel differentially expressed genes.

Metabolic pathways	Gene name	KO	up	down
Auxin signal transduction	ARF	k14486	4	
	GH3	K14487		3
Cytokinin signal transduction	AHP	K14490	1	
	B-ARR	K14491	1	
	A-ARR	k14492	2	
Gibberellin signal transduction	DELLA	k14494	1	
	GID1	k12126	1	
Abscisic acid signal transduction	PYR/PYL	k14496		3
	PP2C	K14497	1	
	SNRK2	K14498	2	
Ethylene signal transduction	ETR	K14509		2
	CTR1	K14510		2
	EBF1/2	K14515	1	
Brassinosteroid signal transduction	TCH4	K14504		1
	CYCD3	K14505	1	
Jasmonic acid signal transduction	COI-1	K13463	2	
	JAZ	K13464		8
Salicylic acid signal transduction	PR1	k13449		2

Due to the enrichment of DEPs in T1 and CK within the peroxisome, there is also enrichment of DEPs in T2 and T1. Additionally, the pathway associated with the DEGs of T2 and CK, along with the DEPs, includes the peroxisome pathway. Therefore, the differential expression of peroxisome channel in *H. rhamnoides* is analyzed here. Among the single up-regulated or down-regulated genes, there were 10 peroxisome-related genes, of which 7 were up-regulated and 3 were down-regulated. As shown in Table 4.

**Table 4.** Differentially expressed genes of *Hippophae rhamnoides* peroxisome channel.

Metabolic pathways	Gene name	KO	up	down
Membrane protein import	PEX19	K13337	1	
Receptor recycling	PEX2	K06664	1	
ROS metabolism	PXMP2	K13347		1
fatty acid-oxidation	HPCL2	K00830	1	
	ACAA1	K07513		1
	PDCR	K11147	1	
sterol precursorbiosynthesis	MVK	K00869		1
amino acid metabolism	AGT	K00830	1	
antioxidant system	SOD	K04565	1	
retinol metabolism	DHRS4	K11147	1	

## Discussion

### *Effects of drought stress on phenotype and enzymatic defense system of Yulu* H. rhamnoides

According to the phenotypic observation, with the deepening of drought stress, the plant height, ground diameter, total biomass, root biomass, and root relative water content of *H. rhamnoides* showed a decreasing trend. Notably, these five indexes were significantly reduced under severe stress (T2) (*p* < 0.05), indicating that the life activities of *H. rhamnoides* were severely limited under severe drought stress with soil moisture content of 4.5%-1.5%. When the soil moisture decreased to less than 1.5%, it may be close to the critical value of soil moisture for the survival of *H. rhamnoides*. When the soil moisture was maintained above 4.5%, the growth and physiological indexes of *H. rhamnoides* were not significantly different from those of CK, and it could maintain normal growth. This finding may inform water management strategies for large-scale cultivation of *H. rhamnoides*. Under drought stress, the total biomass, aboveground biomass and root biomass of *H. rhamnoides* were inhibited, but the root biomass decreased significantly (*p* < 0.05), indicating that the changes of root traits of *H. rhamnoides* were more obvious.

Phenotypic changes are driven by physiological alterations. The POD, SOD and CAT play crucial roles in the enzymatic defense system of plants under stress conditions. These three enzymes work synergistically to eliminate excess free radicals, thereby enhancing the stress resistance of plants.^52^ In general, the activities of POD, SOD and CAT in plants tend to increase under drought conditions, enabling a rapid scavenging free radicals to improve drought resistance. However, the specific changes in the activities of these enzymes can vary among different plant species and levels of drought stress.^53^ In this study, with the deepening of drought, SOD and POD in *H. rhamnoides* roots gradually decreased, and CAT gradually increased. This indicated that drought stress significantly reduced the scavenging capacity of *H. rhamnoides* roots for reactive oxygen species and products of membrane lipid peroxidation, suggesting cellular damage. By increasing the CAT in the roots, Yulu *H. rhamnoides* can more effectively remove the accumulated hydrogen peroxide, thereby protecting the cells from oxidative damage. This action enhances the water transport capacity of the roots, balances the antioxidant enzyme system, and mitigates drought-induced damage to *H. rhamnoides* roots, ultimately improving its drought resistance.

### *Effects of drought stress on gene and protein expression of Yulu* H. rhamnoides

The response of *H. rhamnoides* to drought cannot be fully elucidated solely through phenotypic and physiological changes. The adaptation of plants to drought represents a complex biological process that is regulated by multiple signaling pathways.^54^ With the rapid development of sequencing technology, a large number of drought-related genes have been found. By analyzing these genes and proteins, we gain a molecular framework to enhance plant stress resistance. In order to further investigate the expression of drought-resistant genes and proteins in the roots of *H. rhamnoides* under drought stress, this study employed RNA-Seq and TMT methods for a comprehensive multi-omics analysis.^55^

Through the analysis of the functional enrichment of DEPs and DEGs, it was found that under drought stress, various activities in Yulu *H. rhamnoides*, including hormone regulation, enzymatic reaction, redox processes, transmembrane transport, carbohydrate metabolism, fatty acid, alkaloid synthesis, amino acid metabolism, and nitrogen cycling, were extremely active. Consequently, the plant adjusted its growth strategy, including stomatal opening and closing, osmotic pressure regulation, enzyme activity regulation, water regulation, material distribution, and energy balance. These adjustments enable *H. rhamnoides* roots to maintain the integrity and stability of cellular structure, enhance their defensive capabilities, and alleviate physiological changes caused by drought stress.

This study primarily focuses on the enrichment of DEPs and DEGs, as well as the antioxidant enzymatic defense system. Plant hormone signal transduction pathways and peroxisome pathways were selected for the screening of drought-resistant genes and proteins. Efforts were made to establish a connection between the selected genes and the growth status of sea buckthorn, as well as its antioxidant enzymatic defense system.

### *Effects of drought stress on plant hormone signal transduction channels of Yulu* H. rhamnoides

The plant hormone signaling pathway plays an important role in the drought resistance mechanism of plant roots. This pathway involves the interaction and regulation of various plant hormones to cope with the damage caused by drought stress to plants.^56^ The DEGs between T2 and CK were significantly enriched in this pathway, and a total of 38 genes identified as either single up-regulated or single down-regulated. These genes are instrumental regulating water absorption and utilization, as well as in supporting plant growth and the antioxidant enzyme system under drought stress.

In the process of auxin signal transduction under drought stress, *H. rhamnoides* may enhance the transmission of auxin signals by increasing the expression of ARF and inhibiting GH3 protein, resulting in higher concentrations of auxin.^57^ This mechanism aids in maintaining normal growth and development of plants under drought conditions,^58^ enabling Yulu *H. rhamnoides* to sustain prolonged main root growth, which allows access to deeper water sources and thus improves its moisture absorption and transport capacity to withstand drought stress. In the context of abscisic acid (ABA) signal transduction, this study found that the expression level of PYR/PYL decreased under drought stress, while the upregulation of PP2C and SnRK2 may serve as a regulatory mechanism for *H. rhamnoides* to adapt to the significant reduction in root water content. Previous studies have shown that ABA can promote stomatal closure, reduce water evaporation, inhibit plant growth, and decreases water consumption, thereby maintaining water balance in plants.^59^ In this study, the water content of *H. rhamnoides* roots significantly decreased under severe drought stress. Although the downregulation of PYR/PYL may impact signal transduction during this process, the upregulation of PP2C plays a crucial role by regulating SnRK2 activity, ultimately influencing the development of sea buckthorn’s resistance to stress and minimizing unnecessary physiological activities, thereby conserving resources.

In the process of cytokinin signal transduction, the water content of *H. rhamnoides* roots gradually decreases as drought conditions intensify. Upon receiving drought stress signals, there is an upregulation of AHPs, which accelerates the response to cytokinin signaling.^60^ Additionally, the further mobilization of B-ARR upregulation may promote the transcription of A-ARR genes, enhancing the role of cytokinin signaling in drought resistance.^61^ This mechanism facilitates the elongation of the sea buckthorn root system, thereby increasing the root system’s water absorption capacity and improving drought resistance. During the signal transduction process of gibberellins, the mechanism of action of DELLA protein may be diverse. On the one hand, the expression of DELLA protein is directly affected by drought stress, which can lead to the accumulation and reduce water consumption by limiting the growth of *H. rhamnoides*.^62^ Studies have shown that the degradation of DELLA can promote the growth and development of roots,^63^ enhances the water absorption capacity of plants, and aids in their adaptation to drought stress. On the other hand, DELLA proteins function as nuclear transcription regulators that can inhibit GA signal transduction, thereby restricting plant growth.^64^ Under drought stress, the expression of DELLA-related genes was up-regulated, which inhibited the growth and development of *H. rhamnoides*. However, the up-regulation of GID1 facilitates increased binding of GA and GID1, promoting the degradation of DELLA protein^65^ and relieving the growth inhibition of *H. rhamnoides* caused by drought stress. The results of this study also indicate that, under drought stress, the involvement of DELLA and GID1 significantly reduces plant height, ground diameter, and biomass of *H. rhamnoides*, as the plant adapts to drought conditions by promoting root elongation while inhibiting aboveground growth. This study investigates the unique response of *H. rhamnoides* roots to ethylene signaling under drought stress. Previous research has indicated that ethylene may play a role in the induction of adventitious roots and root hairs.^66^ According to the classical model, EBF1/2 binds to EIN3/EIL1 under stress conditions and facilitates the degradation of EIN3/EIL1 via the ubiquitin-proteasome pathway, thereby negatively regulating ethylene signal transduction.^67^ In our findings, both ETR and CTR1 were down-regulated in *H. rhamnoides* roots, indicating that their activities may be inhibited. The upregulation of EBF1/2 is likely to result in the accumulation of EIN3/EIL1 and an increase in the expression of ethylene-responsive genes. Based on the results of this study, the root biomass and root: shoot ratio of *H. rhamnoides* roots decreased under drought stress, which may be associated with these signaling changes. In the process of brassinosteroid signal transduction, TCH4 is associated with cell wall synthesis and cell growth.^68^ Its downregulation may indicate that the growth of *H. rhamnoides* is inhibited under drought stress, resulting in reduced plant height, ground diameter, and biomass, thereby decreasing water loss. CYCD3 is a key gene in cell cycle regulation, and its up-regulation may promote cell division and proliferation,^69^ aiding *H. rhamnoides* root system in maintaining or increasing cell numbers under drought stress and playing a role during the elongation of the main root.

In the process of jasmonate signaling, the upregulation of COI-1 under drought stress may indicate that *H. rhamnoides* enhance the sensitivity of jasmonate signaling, thereby enabling them to respond and adapt more effectively to arid environments.^70^ Additionally, the down-regulation of JAZ protein may relieve the inhibition of jasmonic acid-responsive transcription factors, allowing these factors to activate the expression of jasmonic acid-responsive genes.^71^ This activation subsequently triggers a series of genes related to drought resistance, such as antioxidant enzyme genes, and osmotic regulator synthesis genes. The dynamic balance between COI-1 and JAZ facilitates sea buckthorn’s ability to better cope with oxidative stress induced by drought by mobilizing genes associated with antioxidant and osmotic regulation. In salicylic acid signal transduction, the down-regulation of PR1 gene may represent a strategy employed by *H. rhamnoides* to respond more effectively to drought stress, reallocating resources and energy to more urgent physiological processes.^72^ In this study, as the degree of drought increased, the root: shoot ratio of *H. rhamnoides* decreased; however, the main root length increased, indicating a redistribution of resources by *H. rhamnoides* under drought stress.

In general, under drought conditions, *H. rhamnoides* utilizes the phytohormone signaling pathway to increase the length of its main root, thereby enhancing its water absorption capacity. This process also optimizes water utilization by reducing plant height, ground diameter, and biomass, while regulating biomass distribution. Additionally, it improves efficiency by regulating the opening and closing of stomata to minimize water evaporation and by modulating the enzyme system to enhance antioxidant capacity, ultimately increasing *H. rhamnoides* drought resistance. The genes involved in the phytohormone signaling pathway primarily influence plant growth and development, with some also being associated with the enzymatic defense system. Moving forward, we aim to further investigate the expression of genes related to the enzymatic defense system.

### Effects of drought stress on peroxisome channels

Peroxisomes contain various oxidases that catalyze the oxidation reactions of diverse substrates, generating hydrogen peroxide in the process.^73^ The expression of peroxisome-related differential genes and proteins may be related to antioxidant enzyme activity, where increasing the activity of these enzymes can enhance the drought resistance of *H. rhamnoides*. In this study, several peroxisome-related genes, including PEX19, PEX2, HPCL2, PDCR, AGT, SOD and DHRS4, were found to be up-regulated, while PXMP2, ACAA1 and MVK were down-regulated. PEX19 is a crucial factor involved in the insertion of peroxisome membrane protein, and its up-regulation may signify *H. rhamnoides* adaptive response to drought stress by increasing the number or activity of peroxisomes.^74^ The upregulation of PEX2 may be associated with the recycling and reuse of membrane proteins.^74^ The upregulation of these proteins indicates that plants are actively enhancing peroxisome function to protect cells from oxidative damage. The upregulation of HPCL2 and PDCR suggests that under drought stress, *H. rhamnoides* enhances fatty acid oxidation, leading to the decomposition of fatty acids for increased ATP production, thereby improving energy utilization efficiency. Additionally, this process elevates the levels of catalase (CAT) in the antioxidant response of *H. rhamnoides* roots. Furthermore, peroxidase (POD) and superoxide dismutase (SOD) work in concert to provide the necessary energy. The upregulation of AGT indicates that under drought conditions, *H. rhamnoides* is modifying its amino acid metabolism to synthesize more stress-responsive proteins or to adjust its nitrogen source utilization. This adjustment is likely in collaboration with catalase (CAT) to eliminate intracellular hydrogen peroxide, thereby preventing cellular damage associated with its accumulation. The upregulation of SOD-related genes may indicate that *H. rhamnoides* is enhancing its antioxidant capacity to cope with the oxidative stress caused by drought, which is essential for protecting cells from oxidative stress damage.^75^ However, the measured value of SOD activity under T1 and T2 drought stress was lower than that of CK. This discrepancy may arise from the degree and duration of drought stress, which can significantly influence SOD activity. Excessive stress may lead to severe damage to plant cells, thereby impacting both the expression and activity of SOD. Furthermore, under drought stress, the reduction in cellular water content may alter the molecular conformation of the enzyme, subsequently affecting its stability and activity. The upregulation of DHRS4 suggests that, in response to drought stress, *H. rhamnoides* may be adjusting its retinol (vitamin A) metabolism to adapt to stress; however, the direct relationship between retinol metabolism and drought stress remains unclear. Alternatively, the upregulation of DHRS4 may help maintain intracellular stability by participating in the synthesis of fatty acids and sterols, as well as scavenging hydrogen peroxide in conjunction with catalase (CAT).

The downregulation of PXMP2 may indicate that, under drought stress, the integrity and functionality of peroxisomal membranes are compromised, leading to a weakened reactive oxygen species (ROS) scavenging ability in sea buckthorn cells. This impairment could result in ROS accumulation and subsequent oxidative stress within the cells. According to the research findings, severe drought stress correlates with decreased peroxidase activity (POD) and superoxide dismutase activity (SOD), suggesting potential damage to the cells of the sea buckthorn root system. Additionally, ACAA1 (acetyl-CoA acyltransferase 1)-related genes were down-regulated under drought stress. While HPCL2 and PDCR were up-regulated under drought stress, the down-regulation of ACAA1 may disrupt normal fatty acid metabolism, potentially impacting energy supply. This metabolic adjustment could be a response to balance energy demand and supply, or it may arise from drought stress affecting specific steps in the fatty acid oxidation pathway. Furthermore, the down-regulation of MVK under drought stress may indicate a reduction in sterol synthesis in *H. rhamnoides*.^76^ This reduction could be attributed to alterations in cell membrane structure and function induced by drought or may reflect a metabolic adjustment aimed at conserving energy and material resources. Overall, the downregulation of PXMP2, ACAA1, and MVK adversely affects the function of peroxisomes, leading to decreased POD and SOD activities, which ultimately restricts the growth and development of *H. rhamnoides*.

Under drought stress, *H. rhamnoides* enhances catalase activity (CAT) through the differential regulation of peroxisome-related genes and proteins, alongside peroxidase (POD) and superoxide dismutase (SOD). Conversely, the activity of *H. rhamnoides* itself declines. This series of reactions suggests that *H. rhamnoides* is attempting to adapt to and mitigate the oxidative stress induced by drought. It appears that *H. rhamnoides* prioritizes the allocation of resources toward increasing CAT activity to better cope with the oxidative challenges posed by drought. In general, these genes reflect an adaptive strategy adopted by *H. rhamnoides* to cope with drought stress. *H. rhamnoides* enhances drought resistance and viability by adjusting its biological processes to optimize resource utilization, improve antioxidant capacity and adjust metabolic pathways.

## Conclusion

Drought stress significantly reduced the plant height, ground diameter, total biomass, root biomass, root relative water content and superoxide dismutase activity (*p* < 0.05). With the increase of drought degree, the main root length and hydrogen oxide enzyme activity of Yulu *H. rhamnoides* showed an increasing trend, and the peroxidase activity showed a decreasing trend, but they were not significant (*p* > 0.05). Soil moisture content remained above 4.5%, Yulu *H. rhamnoides* can grow. Compared with CK, a total of 5789, 5594 differential genes and 63, 1012 differential proteins were obtained in T1 and T2. The joint analysis of transcriptome and proteome showed that the number of DEGs associated with DEPs in T1, T2 and CK was 28 and 126, and 7 and 36 valid KEGG annotations were obtained. Different water conditions affected the gene and protein expression profiles of *H. rhamnoides* roots. Under moderate drought stress and severe drought stress, the differential genes were significantly enriched in the plant hormone signal transduction pathway, but no significant enrichment was found in the protein expression profile. With the increase of drought stress, the combined expression of differential genes and differential proteins increased. Among them, the biosynthesis of secondary metabolites (map01110) of T2 treatment gathered 14 genes. Under severe drought stress, 38 plant hormone signal transduction genes and 10 peroxisome-related genes were screened out, which provided a basis for further study on the molecular mechanism of *H. rhamnoides* roots against drought stress. Yulu *H. rhamnoides* may prioritize the enhancement of CAT activity while simultaneously reducing SOD and POD activities to effectively manage the oxidative stress induced by drought.

## Supplementary Material

Supporting Information__Methods of Proteomic analysis.docx

Supporting Information.xlsx

RNA Seq Power.xlsx

Supporting Information__Fig4_data.xlsx

Supporting Information__Fig3_data.xlsx

Supporting Information__Fig5_data.xlsx

Supporting Information__Fig2_data.xlsx

Supporting Information__figure note.docx

## Data Availability

The RNA-Seq raw sequence data reported in this paper have been deposited in the Genome Sequence Archive in National Genomics Data Center, China National Center for Bioinformation/Beijing Institute of Genomics, Chinese Academy of Sciences (GSA: CRA017824, https://ngdc.cncb.ac.cn/gsa). The proteomics raw sequence data reported in this paper have been deposited in the OMIX, China National Center for Bioinformation/Beijing Institute of Genomics, Chinese Academy of Sciences (OMIX006913, https://ngdc.cncb.ac.cn/omix).
