# Supplementary material for: Growth and physiological response of Yulu Hippophae rhamnoides to drought stress and its omics analysis
Source: Plant Signal Behav. 2024 Dec 9;19(1):2439256. doi: 10.1080/15592324.2024.2439256 (PMC11633206; doi:10.1080/15592324.2024.2439256)
Supplement: Supporting Information__Methods of Proteomic analysis.docx [file KPSB_A_2439256_SM5794.docx]

TMT - Materials and Methods

**1. Total Protein Extraction**

Sample was ground individually in liquid nitrogen and lysed with SDT lysis buffer（4% SDS, 100 mM DTT, 10 mM TEAB）, followed by 5 min of ultrasonication on ice. After reacting at 95 ˚C for 8 min, the lysate was centrifuged at 12000 g for 15 min at 4 ˚C. And the supernatant was reduced with 10 mM DTT for 1 h at 56 ˚C, and subsequently alkylated with sufficient iodoacetamide for 1 h at room temperature in the dark. Then samples were completely mixed with 4 times volume of precooled acetone by vortexing and incubated at -20 ˚C for at least 2 h. Samples were then centrifuged at 12000 g for 15 min at 4 ˚C and the precipitation was collected. After washing with 1mL cold acetone, the pellet was dissolved by dissolution buffer (8 M Urea, 100 mM TEAB, pH 8.5).

**2. Protein Quality Test**

BSA standard protein solution was prepared according to the instructions of Bradford protein quantitative kit, with gradient concentration ranged from 0 to 0.5 g/L. BSA standard protein solutions and sample solutions with different dilution multiples were added into 96-well plate to fill up the volume to 20 μL, respectively. Each gradient was repeated three times. The plate was added 180 μL G250 dye solution quickly and placed at room temperature for 5 minutes, the absorbance at 595 nm was detected. The standard curve was drawn with the absorbance of standard protein solution and the protein concentration of the sample was calculated. 20 μg of the protein sample was loaded to 12% SDS-PAGE gel electrophoresis, wherein the concentrated gel was performed at 80 V for 20 min, and the separation gel was performed at 120 V for 90 min. The gel was stained by coomassie brilliant blue R-250 and decolored until the bands were visualized clearly.

**3. TMT Labeling of Peptides**

Each protein sample was taken and the volume was made up to 100 μL with DB dissolution buffer (8 M Urea, 100 mM TEAB, pH 8.5). Trypsin and 100 mM TEAB buffer were added, sample was mixed and digested at 37 °C for 4h. And then, trypsin and CaCl_2_ were added, sample was digested overnight. Formic acid was mixed with digested sample, adjusted pH under 3, and centrifuged at 12000 g for 5 min at room temperature. The supernatant was slowly loaded to the C18 desalting column, washed with washing buffer (0.1% formic acid, 3% acetonitrile) 3 times, then eluted by some elution buffer (0.1% formic acid, 70% acetonitrile). The eluents of each sample were collected and lyophilized. 100 μL of 0.1 M TEAB buffer was added to reconstitute, and 41 μL of acetonitrile-dissolved TMT labeling reagent was added, sample was mixed with shaking for 2 h at room temperature. Then, the reaction was stopped by adding 8% ammonia. All labeling samples were mixed with equal volume, desalted and lyophilized.

**4. Separation of fractions**

Mobile phase A (2% acetonitrile, adjusted pH to 10.0 using ammonium hydroxide) and B (98% acetonitrile) were used to develop a gradient elution. The lyophilized powder was dissolved in solution A and centrifuged at 12,000 g for 10 min at room temperature. The sample was fractionated using a C18 column (Waters BEH C18, 4.6×250 mm, 5 μm) on a Rigol L3000 HPLC system, the column oven was set as 45°C. The detail of elution gradient was shown in Table 1. The eluates were monitored at UV 214 nm, collected for a tube per minute and combined into 10 fractions finally. All fractions were dried under vacuum, and then, reconstituted in 0.1% (v/v) formic acid (FA) in water.

Table 1 peptide fraction separation liquid chromatography elution gradient table

| Time (min) | flow rate (mL/min) | mobile phase A (%) | mobile phase B (%) |
| --- | --- | --- | --- |
| 0 | 1 | 97 | 3 |
| 10 | 1 | 95 | 5 |
| 30 | 1 | 80 | 20 |
| 48 | 1 | 60 | 40 |
| 50 | 1 | 50 | 50 |
| 53 | 1 | 30 | 70 |
| 54 | 1 | 0 | 100 |

**5. LC-MS/MS Analysis**

For transition library construction, shotgun proteomics analyses were performed using an EASY-nLC^TM^ 1200 UHPLC system (Thermo Fisher) coupled with a Q Exactive^TM^ HF-X mass spectrometer (Thermo Fisher) operating in the data-dependent acquisition (DDA) mode. 1 μg sample was injected into a home-made C18 Nano-Trap column (4.5 cm×75 μm, 3 μm). Peptides were separated in a home-made analytical column (15 cm×150 μm, 1.9 μm), using a linear gradient elution as listed in Table 2. The separated peptides were analyzed by Q Exactive^TM^ HF-X mass spectrometer (Thermo Fisher), with ion source of Nanospray Flex^TM^（ESI）, spray voltage of 2.3 kV and ion transport capillary temperature of 320°C. Full scan range from m/z 350 to 1500 with resolution of 60000 (at m/z 200), an automatic gain control (AGC) target value was 3×10^6^ and a maximum ion injection time was 20 ms. The top 40 precursors of the highest abundant in the full scan were selected and fragmented by higher energy collisional dissociation (HCD) and analyzed in MS/MS, where resolution was 45000 (at m/z 200) for 10 plex, the automatic gain control (AGC) target value was 5×10^4^ the maximum ion injection time was 86 ms, a normalized collision energy was set as 32%, an intensity threshold was 1.2×10^5^, and the dynamic exclusion parameter was 20 s.

Table 2 Liquid chromatography elution gradient table

| Time (min) | flow rate (nL/min) | mobile phase A (%) | mobile phase B (%) |
| --- | --- | --- | --- |
| 0 | 600 | 94 | 6 |
| 2 | 600 | 85 | 15 |
| 78.5 | 600 | 60 | 40 |
| 80.5 | 600 | 50 | 50 |
| 81.5 | 600 | 45 | 55 |
| 90 | 600 | 0 | 100 |

(From Novogene Company (Beijing, China, https://cn.novogene.com/))
