# Supplementary material for: Growth and physiological response of Yulu Hippophae rhamnoides to drought stress and its omics analysis
Source: Plant Signal Behav. 2024 Dec 9;19(1):2439256. doi: 10.1080/15592324.2024.2439256 (PMC11633206; doi:10.1080/15592324.2024.2439256)
Supplement: Supporting Information__figure note.docx [file KPSB_A_2439256_SM5787.docx]

**Fig. 1** Transcriptome differential gene statistics of Yulu *Hippophae rhamnoides*

**Fig.2** KEGG pathways enrichment analysis of DEGs of Yulu *Hippophae rhamnoides*. **A** KEGG pathways enrichment analysis of T1vsCK DEGs of Yulu *Hippophae rhamnoides*. **B** KEGG pathways enrichment analysis of T2vsCK DEGs of Yulu *Hippophae rhamnoides*. **C** KEGG pathways enrichment analysis of T2vsT1 DEGs of Yulu *Hippophae rhamnoides*.

**Fig.3** GO categorization of DEGs of Yulu *Hippophae rhamnoides*. **A** GO categorization of T1vsCK DEGs of Yulu *Hippophae rhamnoides*. **B** GO categorization of T2vsCK DEGs of Yulu *Hippophae rhamnoides*. **C** GO categorization of T2vsT1 DEGs of Yulu *Hippophae rhamnoides*.

**Fig.4** KEGG pathways enrichment analysis of DEPs of Yulu *Hippophae rhamnoides*. **A** KEGG pathways enrichment analysis of T1vsCK DEPs of Yulu *Hippophae rhamnoides*. **B** KEGG pathways enrichment analysis of T2vsCK DEPs of Yulu *Hippophae rhamnoides*. **C** KEGG pathways enrichment analysis of T2vsT1 DEPs of Yulu *Hippophae rhamnoides*.

**Fig.5** Transcriptome and proteome expression regulation. **A** Gene and protein association diagram of T1vsCK. **B** Gene and protein association diagram of T2vsCK. **C** Gene and protein association diagram of T2vsT1. All_tran: all genes identified by the transcriptome; diff_tran: differentially expressed genes identified by the transcriptome; all_pro: all proteins identified by the proteome; diff_prot: differentially expressed proteins identified by the proteome.
